# Lectures on Digestion, Respiration, and Secretion, Given at the Royal Institution, to the Members and to the Pupils of St. George’s Hospital

**Published:** 1851-09

**Authors:** H. Bence Jones

**Affiliations:** Physician to St. George’s Hospital


### RECORD OF MEDICAL SCIENCE.

### ANATOMY AND PHYSIOLOGY.

*Lectures on Digestion, Respiration, and Secretion, given at the Royal Institution, to the Members and to the Pupils of St. George's Hospital.* By H. Bence Jones, M. D., F. R. S., Physician to St. George's Hospital.

### ON THE BILE.

My subject to-day, gentlemen, is the bile, which has, perhaps, given rise to more speculation among physiologists, and more work among chemists, than almost any other which the animal body could furnish. The chemical composition of the bile has formed a subject of inquiry to the very first chemists that have existed, from Berzelius to the present time; but, until very lately, it has not been at all understood. It has also formed the subject of the greatest speculation on the part of the physiologist; and there is scarcely an hypothesis that can be started respecting it which you will not find defended by writers on the physiological properties of the bile. It has been supposed to have the most opposite qualities; and, in fact, there is no limit to the actions which it has been thought capable of effecting. The chemical composition has been most difficult to comprehend, because the bile is a body which can be very easily changed by the different re-agents to which it has been subjected. Even if left to itself, the bile rapidly undergoes change, and new substances are produced from it. Still more rapidly does it change when subjected to chemical re-agents; and hence the opposite statements you meet with in books regarding its composition. It is my object to-day to try and make clear to you, first, the chemical composition of the bile, and, secondly, its physiological properties.

This analysis of the bile shows the kind of substances in it.

*Composition of the Bile.*

| | |
|---|---|
| Water, . . . . . . . . | 90·44 |
| Picromel (gallenstoff) including fat, . . | 8·00 |
| Mucus of the gall-bladder, . . . | 0·30 |
| Extractive common salt and lactate of soda, . | 0·74 |
| Soda, . . . . . . . . | 0·41 |
| Phosphate of soda and lime, and traces of a substance insoluble in alcohol, . . | 0·11 |
| | 100·00 |

You will see from it, that it contains water, salt, and fatty matter; it contains also gallenstoff, a peculiar stuff, of which I shall have to speak in this lecture. The bile may be considered as a compound resembling the soaps, which are combinations of fatty acids and alkalies. The fatty acids have not the same composition as the peculiar acid of the bile, which is combined with soda; but, nevertheless, the bile more closely resembles a soap than any other substance with which I compare it. The most difficult and most complex substance contained in the bile is that peculiar body, gallenstoff, or gall-stuff. It is this which so rapidly undergoes changes, and so readily gives rise to new products,—products which have received so many names, and created so much confusion regarding the constitution of the bile. You may remember, that in a previous lecture, I spoke of a body called "glycocol," or sugar of gelatine. If gelatine was treated with alkali, sugar of gelatine was produced; and you remember, also, that I pointed out, that if benzoic acid was taken, it passed off as hippuric acid, the latter being nothing more than a compound of the elements of benzoic acid and glycocol.

*Relation of Hippuric to Benzoic Acid.*

Hippuric acid = Benzoic acid + Glycocol.

$$C_{18}\ H_8\ N\ O_5 + HO = C_{14}\ H_5\ O_2 + O + {}_4\ H_4\ NO_3$$

It is found, moreover, that glycocol can be abstracted from the bile if rightly treated. This is a most interesting and important fact; and it bears, as you will see, directly upon the composition of the gall-stuff, of which I shall have much to say. The composition of this glycocol is given in this diagram.

*Composition of Substances in the Bile Stuff.*

| | | | | | | |
|---|---|---|---|---|---|---|
| Glycocol, | $C_4$ | $H_4$ | N | $O_3$ | | HO |
| Taurine, | $C_4$ | $H_7$ | N | $O_6$ | $S_2$ | |
| Cholalic acid, | $C_{48}$ | $H_{39}$ | | $O_9$ | | HO |
| Glycochloric acid, | $C_{52}$ | $H_{42}$ | N | $O_{11}$ | | HO |
| Taurochloric acid, | $C_{50}$ | $H_{45}$ | N | $O_{14}$ | $S_2$ | |

Out of the bile, also, can be formed a substance of which you see here, by the kindness of Professor Liebig, a beautiful specimen; it is known by the name of "taurine." It can be obtained without difficulty from the bile; it does not always exist as such in the bile, but can always be obtained from it by re-agents. The most remarkable fact regarding this taurine, is, that it contains much sulphur. You will find that there is another crystalline animal substance, viz., crystine, occasionally found in the urine, which contains the same elements as taurine, only in different proportions. Another substance obtained from bile is called "cholalic acid," of which I have here a specimen in tetrahedral crystals. It is not this acid which combines with soda to form the peculiar constituent of the bile, although it can be obtained from human or ox bile. Cholalic acid has the property of combining with glycocol and forming a compound acid, which may be called "glycocholalic acid." It also forms a compound with taurine, which is called "taurocholalic acid." These two compound acids may be obtained from the bile. The bile is not simply a mixture of taurocholalic acid and glycholalic acid, combined with soda; but these two acids can again combine together to form a still more compound body, which is the peculiar organic constituent of the bile. If this constituent, which has furnished so much work to chemists, be treated in one way, one set of products is formed; and if treated in another way, another set is produced. Hence so many different matters have been said to exist in the bile.

I have here a beautiful specimen of glycocholalic acid combined with potass and soda, forming the crystallized bile of Platner. And in this specimen you may see the glycocholalic acid free, and I have here separate specimens of glycocholalate of soda and glycocholalate of potash.

If bile is decomposed by a very strong acid, and is then left to stand for some time, or even, it is said, if it is left in the human body, and decomposition takes place, a far more insoluble substance, called choloidynic acid, is produced. This acid can also be formed from cholalic acid by boiling it with strong acid; by heat alone the choloidynic acid is converted into an insoluble neutral substance called dyslysin. This acid is probably one of the substances produced in the human body by the bile, in its passage through the bowels. If this acid is oxidised, among other products, it gives precisely the same volatile products as oleic acid. Here is another resemblance between the substances in the bile, and the fatty substances, of which I have spoken in a previous lecture. Thus much for the composition of this peculiar substance existing in the bile.

I trust I have made it clear to you that there exists in the bile taurine, glycocol, and cholalic acid; that they do not exist as such in the bile, but that they form compound acids, like sulphovinic acid, sulpho-saccharic acid, nitro-benzoic and others; they form highly compound or conjugated acids, which can be broken up by the action of different agents upon the bile. But the peculiar constituent of the bile, as a whole, consists of tauro-cholalic acid and glycocholalic acid, combined together, forming a double conjugate acid, which is united with soda.

A peculiar test for the bile has been given us by Pettenkoffer, which, for its beauty, well deserves to be shown to you. If I take a little ox-bile, freed from all mucus, (which can be done by means of alcohol,) mix it with water, and then add one drop of sugar and a little sulphuric acid, free from sulphurous, I shall have what is known as Pettenkoffer's test for the bile. The strength of the sugar should be one part of sugar to four parts of water. A yellow precipitate is first formed; it then deepens in color, as you see, until it becomes red, and at last a beautiful crimson. The heat produced by the mixture of the acid with the water is generally sufficient for the test. Instead of sugar, acetic acid, and probably other substances may be used.

There is another peculiar substance in the bile which deserves your attention; it is known by the name of cholesterine. It differs somewhat in its re-actions from ordinary fatty matter. I have here a beautiful specimen of this cholesterine in crystals. The substance has this re-action: it is soluble in alcohol by the aid of heat, and when cooled it crystallizes out in beautiful plates. It is a substance containing carbon, hydrogen, and oxygen, and may be represented by the formula $C_{81} H_{69} O_8$. It is easy to be determined, from its crystalline form and chemical re-actions; fusing most easily, and burning with a smoky flame. Cholesterine forms the usual ingredient of most biliary calculi. It is rarely that the highly complex acids of which I have spoken enter into the composition of biliary calculi; but the cholesterine, from its insolubility, is much more apt to do so; in fact, but for this substance, gall-stones would be exceedingly rare. Taurocholalic acid dissolves the cholesterine very easily, but glycocholalic acid scarcely at all. Taurocholalic acid is very probably the solvent of the cholesterine in the bile. If in the gall-bladder the taurocholalic acid should undergo any change,—if there should be any loss of its properties, or any conversion of it into another substance, (and it easily undergoes change,) then the proper solvent for the cholesterine is gone, and the cholesterine falls as a precipitate. Very frequently in gall-stones it is found that a small particle of coloring matter forms the centre on which the cholesterine crystallizes. I have here many thousands of biliary calculi removed from one patient; and mixed with the gravel are little particles of dark green biliary matter. Moreover, little particles of coloring matter may be seen to form the nucleus of each little stone, on which the cholesterine has crystallized; sometimes cholesterine calculi attain the enormous size of the magnificent specimen belonging to this institution. Cholesterine is not peculiar to the bile; in this long tube you see crystals of cholesterine removed from a tumor. It exists in the brain, and is formed in atheroma and in chronic abscesses, in tubercles and in pus.

Besides these substances, the bile contains salts; that is, if burned, it gives ashes; and it is found that healthy bile is always of an alkaline reaction, from the quantity of soda present in it. Lastly, the quantity of water contained in the bile is very considerable, but by no means so much as we found in the gastric juice and the saliva; its specific gravity amounting to 1026 or 1030.

Regarding the coloring matter much has been said, but little work has been given to it except by Berzelius, who states that the coloring matter of the bile and the coloring matter of grass are identical; and that this is so, whether the bile comes from vegetable or animal feeders. This coloring matter, perhaps, is a most important accessory, of which we do not know the physiological importance. On the other hand, it may be only a substance derived from the coloring matter of the blood, after it has served the purposes for which it was formed. Time only admits of my stating that, as far as the examination of Berzelius goes, the coloring matter of the bile and that of the grass are both chlorophyl, and are therefore perfectly identical.

I come now to the quantity of the bile formed. This probably varies very much in different animals and in different human beings. Of course experiments cannot be made as to the quantity of bile in human beings; animals alone become subjects for research in this respect. It has been found, that a cat weighing 2¼ lbs. during the most perfect digestion, secreted 11·8 grains of bile in an hour. It appears from experiments in Germany, that the quantity of bile secreted reaches its height about ten or twelve hours after food; that it then diminishes to what it was an hour or two before food. This is a most important fact regarding the physiology of the bile—that it requires as much as ten hours after food has been taken for it to reach its highest quantity:—

*Quantity of Bile at different hours after Food.*

| | | | Quantity of Bile. |
|---|---|---|---|
| Cat after eating flesh, | 2nd hour | . | 7·5 grs. |
| " | 4th " | . | 9·7 |
| " | 6th " | . | 11·6 |
| " | 8th " | . | 12·7 |
| " | 10th " | . | 13·0 |

From the 10th to the 24th hour it diminished at the rate of ·4 of a grain per hour.

By continued starvation the quantity of bile decreases; and in perfect starvation no bile passes out of the gall-bladder at all, though the bladder is always distended with bile. In cases of starvation in human beings, which unfortunately sometimes occur, the gall-bladder is found full of bile, though none can be found in the intestines.

The most remarkable discovery which has lately been made with regard to the bile has been given to us by M. Bernard, a French physiologist, of whom I spoke in a previous lecture, on the pancreatic fluid; the discovery is perfectly original, and deserves the very greatest praise; and it will probably be found one of the most important with which we have lately been made acquainted.

Not only does the bile of those who feed on starch and vegetables contain sugar, but sugar is found also in animals that feed on meat only. During the period of digestion the liver always contains much sugar. M. Bernard has found three times, even in man, that this is the case. I have here an extract from the liver of one of the persons on whom he made his experiments. The sugar cannot be long in the liver without undergoing change,—being converted perhaps, into lactic, or some other acid. Ten or twelve hours after the death of a man or an animal, no sugar would be found in the liver. One liver experimented upon by M. Bernard was obtained from a woman who was executed in Paris for murder. He found that an extract obtained from this liver manifestly contained sugar; and I hope to be able to show you this on a portion of this extract, as he showed it to me.

If I boil this extract in water, I shall dissolve the sugar, if there be any present, and then I shall have a solution to which I can apply my test—that of sulphate of copper and liquor potassæ, by which a reduction will instantaneously take place, such as you have frequently seen. Here is the rapid reduction. M. Bernard finds that in all kinds of animals—mammifers, birds, reptiles, fish, and molluscs—the liver, during digestion, contains sugar. By continuous abstinence, in warm-blooded animals, very much of the sugar disappears. During the period of digestion, the blood which passes out of the liver by the hepatic vein always contains a very considerable quantity of sugar, whatever the nutriment may have been. This is different, perhaps, from what we might have expected at first sight. We might have supposed that the sugar would be absorbed by the veins, and as the blood passed into the liver, we should have much more sugar than as it passed out. M. Bernard found that dogs kept on flesh alone for three, four, five, six, or eight months, gave no sugar in the blood passing into the liver by the vena porta, but in the blood passing out of the liver, sugar was distinctly present. Lehmann, a German chemist of great authority, has also found, in five different horses, from ten to sixteen times as much sugar in the blood that passed out of the liver, as in that passing into the liver. When the animal was fasting, the blood of the vena porta gave no sugar. The hepatic vein gave it easily and distinctly. Not only did he detect sugar by the reduction of the copper,—which reduction might be said to arise from some other cause,—but he found that it could be fermented, and that carbonic acid and alcohol could be thus obtained. Sugar has also been found in the liver of the fœtus.

M. Bernard says that the function of the liver is twofold; one, the formation of bile, which is thrown out; and the other, the formation of sugar, which is kept in. He has gone still further, and has endeavored to establish the influence of the nerves in the production of sugar. He thinks that the section of the pneumo-gastric nerves near the heart causes the sugar to disappear from the liver altogether; and that by exalting the action of this nerve, and especially by irritating it at its origin in the brain, the quantity of sugar thrown out can be greatly increased. I shall have occasion to return to this fact in my lecture on diabetes. However this may be, there can be no doubt that by the change which the blood undergoes in the liver, sugar is actually produced. The facts are these. Much fatty matter is found going into the liver by the portal vein with but little sugar; and from the hepatic vein there comes out much sugar, with but little fat. The explanation of this has not yet been fully ascertained. A conjecture has been given by one who always abounds in valuable conjectures—M. Schmidt, a German physiologist. I may show you this possible conversion of fat into sugar, thus:

*Relation of Fat to Bile.*

$$\text{Fat acid} + 14 \text{ oxygen} = \text{cholalic acid} + 8 \text{ water}$$

$$C_{48} H_{47} O_3 + 14\ O = C_{48} H_{39} O_9 + 8\ HO$$

$$2 \text{ eq. glycerine} + 4 \text{ oxygen} = \text{sugar} + 2 \text{ water}$$

$$C_{12} H_{14} O_{10} + 4\ O = C_{12} H_{12} O_{12} + 2\ HO$$

Suppose that going into the liver we have fat, consisting of fatty acid and glycerine, and that coming out of the liver, we have bile and sugar; then supposing the process of oxidation to take place—that is, that the fatty matter obtains oxygen—we have fatty acid and 14 eq. of oxygen, which give the elements of cholalic acid and water; whilst from glycerine with oxygen we have the elements of sugar and water. Thus, if we suppose that in the bile a process of oxidation is taking place, we have an explanation of the fat going in and the sugar coming out. I give this simply as a conjecture, I do not say that it is so; for no such change has been effected out of the human body by acting on fat. It is, at any rate, an explanation which enables us to remember the fact, that fatty acid and glycerine go into the liver, and that coming out of it, we have bile acid, and sugar. The bile acid is thrown out, and is used for purposes of which I shall have to speak; and the sugar passes in, continues in the circulation and is used for further purposes in the animal economy.

What, then, is the physiological action of the bile? The most opposite and the most important actions have been attributed to it. It has been said to promote digestion, and to stop digestion. Some say that it neutralises free acid, thus lessening irritation; others, that it increases the peristaltic action of the bowels, thus increasing irritation. It has been said to be partly absorbed into the system to support respiration, by furnishing a highly carbonaceous body. Some have said that it promotes the absorption of fatty substances; and by others, it has been said to have no action upon fats at all. To solve these questions was the difficulty. Experiments were tried by tying the common duct through which the bile passed; but this is not the way to arrive at a satisfactory result. If the bile is not suffered to pass, a stoppage is put to the functions of the liver; the whole order of the system is thrown out, and general disorder is produced. In 1844 a new mode of experimenting was begun by Schwann, who collected the bile without allowing it to pass into the intestines, by means of an opening similar to that which I mentioned in the case of the pancreatic duct. The action of the liver thus went on as usual, and all the functions of the body were performed without impediment. Twelve dogs lived from sixty-four to eighty days without any bile passing into the intestines; one dog, thus experimented upon, lived four months; and another, belonging, I believe, to M. Bernard, lived a year in this state. It was found that dogs thus treated ate much, and digested badly, partly in consequence of the unnatural fistulous opening. They did not lose much weight at first; but after a little time they lost their appetite, became thin, and ultimately died. The bowels acted as regularly and perfectly as if the bile had passed in the usual manner. Professor Nasse had a dog that lived from the 12th of August to the 27th of January. The quantity of bile varied with different kinds of food between 31 grains and 370 grains daily, with from 16·44 to 19·19 per cent. of solid constituents. Less was secreted when the dog was ill. The dog ate much; digested badly; did not lose weight at first; afterwards lost its appetite, and then became thin. M. Blondlot had a dog that flourished for three months. The bowels acted twice daily.*

Even in human subjects, it has been found that when a fistulous opening has been made, owing to perfect obstruction of the common duct, by inflammatory action, the bowels have continued to act when the bile did not pass,—showing that the bile is by no means indispensable for their action.

Many experiments were tried with dogs, as to the quantity of bile secreted. The influence of medicine was also tried; and it is interesting to us to know that the action of mercury was decidedly to increase the quantity of bile secreted, as has long been held by medical men. If animals can live for a year, enjoy tolerable health, and digest their food,

*At the meeting of the French Academy, on the 23rd of June this year M. Blondlot gave the history and *post-mortem* of a dog that lived for five years without bile passing into the intestines.

without any bile passing into the intestines, the importance of bile and its necessity for the purposes of digestion have been exaggerated.

The action of bile out of the body on the different constituents of food, tends to precisely the same results as we have seen obtained by experiments in the body. Bile, when mixed with neutral fat or with oil, is found to have no chemical action whatever. It makes a sort of emulsion only, not quite so good as that produced by the pancreatic fluid. I added to solutions both of pancreatic juice and bile, equal quantities of water and oil, and then left them, after agitation for some time, to see which produced the most enduring emulsion. You see them here; both have caused the fatty matter to be minutely divided: but I think the pancreatic fluid has divided it and kept it divided the best. When fresh out of the body bile has no action on starch; it does not change it into sugar, as we saw the saliva did. When, however, it is allowed to decompose, it has a slight action upon starch; but not more than all animal substances have. It has no action on cane-sugar until after it has stood for a considerable length of time, and then the cane-sugar is converted into acid. With grape-sugar, if left for any length of time, it forms lactic acid; but so do all other animal substances when in contact with sugar. It has no action, even when acidulated on casein, or on the albuminous substances which constitute our food.

It has been said that the liver purifies the blood by removing a large quantity of carbonaceous substance from it. To determine this by absolute experiment was a matter of great difficulty; but Schmidt has endeavored to solve this question by experiment on forty cats, thirteen geese, many sheep and rabbits, in which he made fistulous openings into the gall-ducts for the purpose of collecting all the bile, and of determining the proportion between the quantity of carbonic acid thrown out by the lungs and the quantity of carbon in the bile. He passed a tube into the gall-duct, and could measure how much gall came out per hour; and he could determine the composition of the bile by burning it and collecting the carbonic acid. He made, at the same time, comparative experiments on the respiration, some of which I shall have to detail to you in a future lecture; and he came to the conclusion that not more than from one-tenth to one-fortieth of the carbon which passes out of the body passes by the liver, and that therefore the liver has no considerable action in freeing the blood from carbonic acid or carbon. He found that eight-ninths or nine-tenths of the carbonaceous matter remains in the circulation, and does not pass out by the bile at all, but is thrown out through the lungs; a small portion, however, must escape in the urine, probably not much less than passes out in the bile. But I am unable to give you the proportion of carbon in the urine and bile daily excreted, from want of experiments.

What, then, in conclusion, are the uses of the bile? I have shown you that it is an alkaline fluid, and a body resembling soap. If soap is brought into contact with an acid, you know what happens: the alkali of the soap and the acid combine, and the acid of the soap is set free and precipitated. So, also, is it in the bile. If I take human bile, and mix it with acid, (as you see in the experiment, with sulphuric acid,) a greenish white precipitate is formed. Let me show you what would happen to human bile, if mixed with the acid secretion of the stomach. This I can do by adding dilute hydrochloric acid to a portion of bile, or better still, by mixing some of the clear fluid obtained from the contents of the stomach, which I showed you in my lecture on the gastric juice; by both a precipitate will be immediately produced. (Experiment.) The alkali which exists in the bile goes to the acid; it neutralizes so far, the acid reaction coming from the stomach; and it precipitates the insoluble acids, which give rise to choloidynic acid, and even to that still more insoluble substance, Dyslysin, in its passage through the intestinal canal. It appears to me, then, that one great action of the bile is to furnish an alkaline fluid, which, when mixed with the acid secretion that has served the purpose of dissolving the albumen, will neutralize it, and lessen its acidity, so as to prevent it from producing irritation and increased action of the intestinal canal. That the stomach can actually bear much stronger acid than the bowels, is known to most medical men. That the acid does not pass rapidly out of the stomach I am convinced by the following experiment. To an adult man I gave 162 grains of dry, pure tartaric acid dissolved in two ounces of water. No pain was felt for three hours; no food was taken during this time; and without doubt, all the tartaric acid would in these three hours have been absorbed, or would have passed out of the stomach. At the end of this time, a pain in the bowels began to be felt, and at the end of the fourth hour there was very considerable pain, coming on in paroxysms. At the lapse of about five hours, if the bowels had been allowed to act, they would have acted from the acid thus taken. A repetition of the experiment, with 84 grains, gave precisely the same results. When the acid entered the bowels, pain began to be felt, and, if bile in plenty had been poured out, the acid would have been neutralized, in part at least; the alkali would have combined with the acid; the insoluble bile acid would have been formed as a precipitate, and been thrown out of the body. If this be so, sluggishness of the liver, a deficiency of alkali poured into the duodenum, becomes a reasonable cause of excessive acidity of the intestines; the gastric acid required to dissolve the albuminous food, if sufficient bile is not formed, will pass into the intestines, and produce irritation and increased action. The physician has long held, that want of action of the liver gives rise to acidity, and that alterative medicines correct this state.

But the very great size which the liver attains in the fœtus appears to indicate that it performs some additional action independent of food and of digestion. This additional action has been said, by German physiologists, to be the reparation and the formation of blood globules; but this is by no means proved. It seems to me much more probable that it is for the purpose of neutralizing the acid, and probably also, for the purpose of removing, when requisite, some of the carbonaceous substances; in certain states compensating for the action of the lungs, though, in ordinary states, removing much less carbon than has been said. The bile gives water, moreover, to dilute the chyle; it tends to the subdivision, in some degree, of the fat and the oil of our food. It acts upon the free acid of the intestine; and some of it may be possibly absorbed, and pass into circulation again, as Professor Liebig originally conjectured. It is not nearly so important as the gastric juice, which dissolves the albuminous part of our food, or the pancreatic fluid, and the salivary fluid, which convert all the insoluble starch, as I have shown you, into soluble sugar. Lastly, the importance of the bile in forming sugar from fat, is one of those facts which cannot be overrated. By this discovery of M. Bernard's, very important knowledge relating to the physiology and pathology of man will be obtained during the next few years; at least there can be little doubt, that the disease known as diabetes, if not closely connected with this production of sugar in the liver, must at least be influenced by it to a very considerable extent.—*London Medical Times.*

